# Shelter Dogs as Sentinels for *Trypanosoma cruzi* Transmission across Texas

**DOI:** 10.3201/eid2008.131843

**Published:** 2014-08

**Authors:** Trevor D. Tenney, Rachel Curtis-Robles, Karen F. Snowden, Sarah A. Hamer

**Affiliations:** Texas A&M University, College Station, Texas, USA

**Keywords:** Chagas disease, *Trypanosoma cruzi*, Texas, hematophagous triatomines, kissing bug, vectorborne, parasite, autochthonous, enzootic, parasites, *Suggested citation for this article*: Tenney TD, Curtis-Robles R, Snowden KF, Hamer SA. Shelter dogs as sentinels for *Trypanosoma cruzi* transmission across Texas. Emerg Infect Dis. 2014 Aug [*date cited*]. http://dx.doi.org/10.3201/eid2008.131843

## Abstract

Chagas disease, an infection with the parasite *Trypanosoma cruzi*, is increasingly diagnosed among humans in the southern United States. We assessed exposure of shelter dogs in Texas to *T. cruzi*; seroprevalence across diverse ecoregions was 8.8%. Canine serosurveillance is a useful tool for public health risk assessment.

The protozoan parasite *Trypanosoma cruzi* is the causative agent of Chagas disease, a neglected tropical disease affecting >8 million persons across Mexico and Central and South America. In the United States, estimates of human infection range from 300,000 to >1 million (*1**,**2*). Although immigrants exposed in Chagas disease-endemic regions constitute the majority of infected persons in the United States, autochthonous transmission is increasingly recognized (*3*), and enzootic cycles involving infected wildlife reservoirs and domestic dogs occur across the southern United States. (*4*). Vectorborne transmission occurs through contamination of the bite site or mucous membranes with feces of infected hematophagous triatomines (“kissing bugs”). In addition, the parasite can be passed through consumption of infected bugs or contaminated food products, through blood transfusions, and congenitally (*4*).

Clinical manifestation in humans and dogs ranges from asymptomatic to acute myocarditis and sudden death to chronic progressive cardiac disease (*5**,**6*)*.* No vaccine is available for humans or dogs. Drugs used to treat Chagas disease in humans have not been approved by the US Food and Drug Administration and are available in the United States only through investigational protocols. The disease is notifiable in 4 states including Texas, where as of 2013, human and veterinary cases must be reported.

Texas is a high-risk state for transmission of *T. cruzi* to dogs, considering the diversity of triatomine vectors, reservoir hosts, and previous documentation of canine disease (*5**,**7*). Because dogs arriving at shelters may have high exposure to vectors, we expect that shelter dogs will provide a sensitive spatial index of Chagas disease risk across the landscape. The objective of this study was to measure *T. cruzi* seroprevalence in shelter dog populations across Texas.

## The Study

To assess exposure to *T. cruzi*, we established a network of 7 canine shelters in major cities and rural areas representing diverse ecoregions across Texas (*8*; Figure). Using a cross-sectional study design, we collected blood samples during May–August 2013 from ≤30 dogs at each shelter, in adherence with client-owned animal use protocols approved by Texas A&M University Institutional Animal Care and Use Committee. Enrollment criteria for our study included dogs >6 months of age within 4 days of admittance to shelters. We detected exposure to *T. cruzi* using Chagas STAT-PAK, a commercially available rapid immunochromatographic test (Chembio Diagnostic Systems, Inc., Medford, NY, USA) that has been validated for use in dogs (*9*) and has high sensitivity and specificity when compared with conventional serologic techniques (*10*). To detect parasite DNA within blood, we followed this process: a 200-μL aliquot of centrifuged blood, including the buffy coat, from a subset of seropositive and seronegative dogs across all shelters was subjected to DNA extraction and used as the template in a real-time quantitative PCR with a TaqMan probe to amplify a 166-bp repetitive sequence of satellite DNA specific to *T. cruzi* (*11*). Using a sequential testing approach, we subjected positive samples in the real-time quantitative PCR to a second confirmatory PCR using previously published TCZ primers to amplify a 188-bp sequence of satellite DNA (*12*). Samples positive by both assays were considered to contain *T. cruzi* DNA within the blood.

**Figure:**
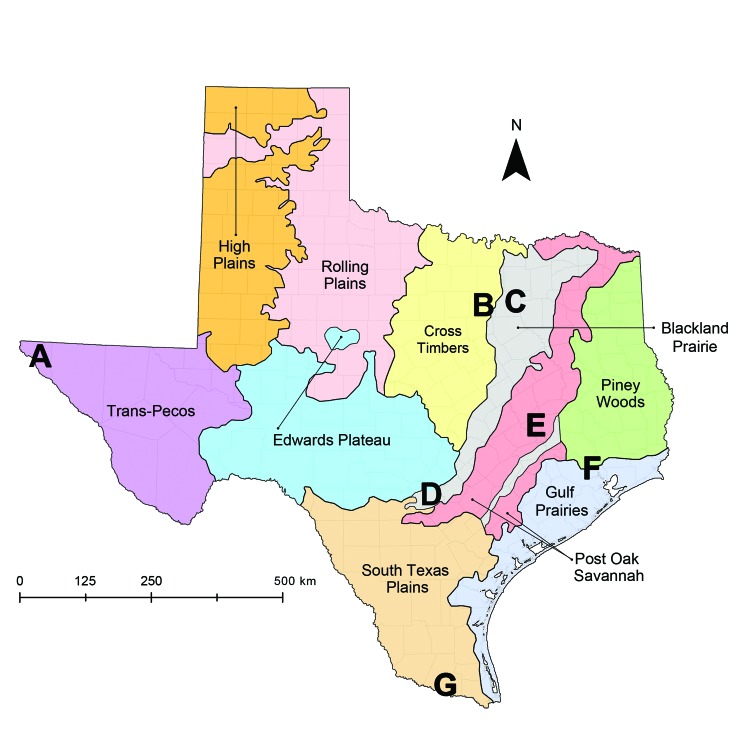
Locations of canine shelters within Texas, United States, 2013. Shelters (A–G) are distributed across 7 of the 10 Gould Ecoregions of Texas (*9*), Map obtained from Texas parks and Wildlife Department (http://www.tpwd.texas.gov/publications/pwdpubs/media/pwd_mp_e0100_1070ad_08.pdf).

A total of 205 blood samples were collected from shelter dogs. Dogs enrolled in the study ranged from 6 months to 13 years of age and represented diverse breed groups. A total of 18 (8.8%) dogs were seropositive for *T. cruzi* antibodies. Seropositive dogs were found at all shelters within the network; shelter prevalence ranged from 6.7% to 13.8% (Table). Using logistic regression, we found no differences in the odds of seropositivity across location, sex, age category, breed group, and dog origin (Table). In a subset of 50 dogs, including 14 seropositive and 36 seronegative dogs, 3 (6%) blood samples were positive for *T. cruzi* DNA by both PCR methods, each with similar cycle threshold values indicative of ≈50 parasite equivalents of DNA per mL of blood. PCR-positive dogs included 2 male and 1 female dog of sporting and toy breeds from shelters A, C, and F, ranging in age from 1.5 to 7 years. Of these 3 dogs, 1 was serologically positive for antibodies against *T. cruzi*.

**Table:** Logistic regression model for risk for *Trypanosoma cruzi* seropositivity from 205 dogs across Texas, 2013

Risk factor	No.*	No. seropositive (%)	Odds ratio	95% CI	p value
Location (Figure label)					
El Paso (A)	29	2 (6.9)	0.69	0.11–4.47	0.69
Fort Worth (B)	30	2 (6.7)	0.67	0.10–4.30	0.67
Dallas (C)	30	3 (10.0)	1.04	0.19–5.59	0.97
San Antonio (D)	29	4 (13.8)	1.49	0.30–7.33	0.62
College Station (E)	31	3 (9.7)	Referent	Referent	Referent
Houston (F)	26	2 (7.7)	0.78	0.12–5.05	0.79
Edinburg (G)	30	2 (6.7)	0.67	0.10–4.30	0.67
Sex					
F	105	9 (8.6)	Referent	Referent	Referent
M	96	9 (9.4)	1.10	0.41–2.95	0.84
Age†					
<2 y	115	8 (7.0)	Referent	Referent	Referent
>2 y	84	10 (11.9)	1.81	0.68–4.94	0.23
Breed group‡					
Herding and working	53	5 (9.4)	Referent	Referent	Referent
Hound, nonsporting, and toy	43	4 (9.3)	0.98	0.23–3.96	0.98
Sporting	44	6 (13.6)	1.52	0.43–5.62	0.52
Terrier	56	1 (1.8)	0.17	0.01–1.13	0.12
Origin					
Owner surrender	40	3 (7.5)	Referent	Referent	Referent
Stray	137	10 (7.3)	1.08	0.32–4.94	0.91
Transfer from another shelter	21	4 (19.0)	2.9	0.58–16.13	0.19

*Sample size for some variables does not equal 205 because complete information was not available for all dogs. †Age category was estimated on the basis of patterns of dentition and tooth wear. ‡Most dogs were mixed breed on the basis of appearance; American Kennel Club breed group assignment is based on dominant breed features.

Additionally, we observed very faint-colored bands on serologic dipsticks in samples from 26 dogs that are not included in the overall seroprevalence estimate. Because we were uncertain of how to interpret antibody presence in these samples, we submitted a subset (n = 11) for indirect fluorescent antibody testing at the Texas Veterinary Medical Diagnostic Laboratory, of which 4 samples (36.3%) tested positive. Although cross-reactivity cannot be ruled out by using the indirect fluorescent antibody technique, these data suggest that the seroprevalence we report (8.8%) is a conservative estimate.

## Conclusions

Shelter dogs had widespread exposure to *T. cruzi* across 7 ecologic regions in Texas, with a conservative statewide average of 8.8% seroprevalence. The presence of seropositive dogs across all sampled regions, age classes, breed groups, and canine origins suggests that ecologic requirements for parasite transmission to dogs are not constrained to focal areas or particular breed groups. Although the travel histories of dogs in our study are unknown, the presence of antibodies in dogs across all age classes, including young dogs that are less likely to have traveled, suggests local exposure. Furthermore, at least some shelters are located in regions in which kissing bugs have previously been reported (*7*). The only published prospective seroprevalence study of dogs from 1 county in south Texas reported a seroprevalence of 7.5% (n = 375 stray dogs) (*13*), similar to our statewide average. Across 2 regions in Mexico, including 1 where Chagas disease was previously considered nonendemic, seroprevalence of *T. cruzi* in canines ranged from 17.5% to 21% and was directly correlated with *T. cruzi* seroprevalence in humans in these regions (*14*). Further research is needed to quantify the association between infection of canines with *T. cruzi* and risk for Chagas disease among humans in the United States.

The widely accepted concept of Chagas disease is that *T. cruzi* infection is lifelong, which is supported by continuous detection of antibody presence within hosts. Despite detection of antibodies in dogs sampled across the state, parasite DNA was detected in the blood of only 3 dogs. Limited experimental investigations of *T. cruzi* infection of dogs indicate that parasitemia is detectable cytologically as soon as 3 days after inoculation and lasts <3 weeks (*5*), after which the parasite localizes in tissues and parasitemia is undetectable. Our observations suggest that most seropositive dogs were not in the acute phase of the infection at the time of sampling. Although domestic dogs have been shown to serve as reservoir hosts in some regions of Central and South America (*15*), the importance of dogs relative to wildlife hosts as reservoirs in the United States is unknown.

Dogs that arrive at shelters, especially stray dogs, are likely to have had increased exposure to the outdoors and vectors and have been shown to have higher exposure to vectorborne pathogens than client-owned dogs that are brought to veterinary clinics (*16*). Shelter dogs, therefore, provide a sensitive population for assessment of local canine transmission risk, and we suggest that awareness of kissing bugs and Chagas disease risk among citizens and the medical community should be heightened in areas where seropositive dogs are detected.
